# Association of labor epidural analgesia with early postpartum pelvic floor function in primiparous women undergoing vaginal delivery: a retrospective cohort study

**DOI:** 10.3389/fsurg.2026.1910839

**Published:** 2026-08-21

**Authors:** Lishan Lin, Yidan Wang, Yaping Wang, Mengjie Yan, Mingting Wang, Suoqin Ge, Junsheng Zhu, Maohong Gu

**Affiliations:** 1 Department of Obstetrics and Gynecology, Nanjing First Hospital, Nanjing Medical University, Nanjing, Jiangsu, China; 2 Department of Ultrasound, Nanjing First Hospital, Nanjing Medical University, Nanjing, Jiangsu, China; 3 Department of Anesthesiology, Nanjing First Hospital, Nanjing Medical University, Nanjing, Jiangsu, China

**Keywords:** labor epidural analgesia, pelvic floor function, postpartum recovery, primiparous women, vaginal delivery

## Abstract

**Background:**

The association between labor epidural analgesia (LEA) and postpartum pelvic floor function remains uncertain. We evaluated whether LEA was associated with pelvic floor outcomes in primiparous women undergoing vaginal delivery.

**Methods:**

This single-center retrospective cohort study included 200 primiparous women with singleton, cephalic vaginal deliveries. Participants were classified according to LEA exposure. Baseline and peripartum characteristics were compared in the overall cohort. Early postpartum pelvic floor outcomes at 6–8 weeks were analyzed in 191 women, and multivariable regression was performed in 175 complete cases. Outcomes included type I and type II pelvic floor surface electromyography, bladder neck mobility, posterior bladder angle during Valsalva, levator hiatus area during Valsalva, urinary incontinence, and pelvic organ prolapse. The multivariable models additionally adjusted for second-stage labor duration and oxytocin use, which were considered potential labor-process variables in the interpretation of the adjusted estimates.

**Results:**

No statistically significant differences were identified in the measured baseline characteristics between the LEA and non-LEA groups. Compared with non-LEA, LEA was associated with longer first-, second-, and third-stage labor, longer total labor duration, and more frequent oxytocin use. No significant detectable between-group differences were observed in any pelvic floor parameter, urinary incontinence, pelvic organ prolapse, prolapse stage, or levator avulsion. After adjustment for maternal age, pre-pregnancy body mass index, gestational age, neonatal birth weight, second-stage labor duration, and oxytocin use, LEA was not significantly associated with any continuous pelvic floor outcome, urinary incontinence (adjusted odds ratio, 1.305; 95% confidence interval, 0.629–2.710), or pelvic organ prolapse (1.771; 0.717–4.373).

**Conclusions:**

LEA was associated with altered labor characteristics, whereas no statistically detectable association with worse pelvic floor outcomes at 6–8 weeks postpartum was observed after adjustment for the measured covariates. Given the observational design, potential residual confounding, and limited precision for infrequent outcomes, modest associations cannot be excluded.

## Introduction

Labor epidural analgesia (LEA) has become one of the most commonly used and most effective pharmacological methods for pain relief during childbirth and is now embedded in standardized intrapartum management and obstetric care pathways (1–3). However, although its analgesic benefit and contribution to maternal childbirth experience are well recognized, whether LEA affects postpartum pelvic floor recovery remains inconclusive (4). On the one hand, previous studies have suggested that LEA may be associated with prolonged first- and second-stage labor, and some investigations have further reported increased rates of instrumental delivery or early postpartum urinary incontinence, supporting the biological plausibility that LEA could influence pelvic floor recovery indirectly by extending pelvic floor loading and increasing delivery-related mechanical stress (5, 6). On the other hand, earlier retrospective studies, randomized controlled trials, and prospective cohort studies did not demonstrate clear deterioration in postpartum pelvic floor electromyographic function, manometric parameters, or most pelvic floor disorder symptoms among women receiving neuraxial labor analgesia, suggesting that the true effect of LEA on pelvic floor outcomes may not be uniform or unidirectional (6–9). Therefore, in the context of the widespread use of LEA, further clarification of its relationship with postpartum pelvic floor recovery is of substantial theoretical and clinical importance and may help optimize analgesic decision-making, peripartum counseling, and early postpartum pelvic floor screening strategies (4).

Pelvic floor disorders (PFDs) are not limited to a single symptom but comprise a spectrum of clinical conditions, including urinary incontinence, pelvic organ prolapse, and abnormalities in pelvic floor support structures as well as neuromuscular function; from both epidemiological and life-course perspectives, pregnancy and childbirth—particularly vaginal delivery—are among the major risk factors for their development and progression (10). In the early postpartum period, pelvic floor injury often first manifests as objective alterations, such as reduced muscle strength and endurance, impaired support, increased bladder neck mobility, or enlarged levator hiatus dimensions; these changes may precede overt symptoms and may already indicate an increased risk of subsequent stress urinary incontinence or prolapse, meaning that symptom-based evaluation alone is insufficient to fully characterize pelvic floor recovery (11, 12). Longitudinal evidence further suggests that postpartum pelvic floor recovery is not immediate, with most structural and functional restoration occurring during the first 6 months after delivery, although not all women return to their pregnancy baseline, making the 6–8-week postpartum period a clinically meaningful window for early identification of abnormalities, assessment of recovery trajectories, and initiation of targeted intervention (12). In addition, objective assessment tools, including pelvic floor ultrasound, MRI, and electrophysiological testing, have been shown to capture childbirth-related structural and functional changes and can therefore support early screening, risk stratification, and subsequent rehabilitation planning (13). Accordingly, when evaluating the potential impact of LEA on postpartum recovery, early pelvic floor function represents a highly relevant outcome with both pathophysiological and clinical significance.

Although the body of literature addressing the relationship between LEA and postpartum pelvic floor outcomes has grown, important limitations remain in terms of study populations, outcome selection, follow-up windows, and control of confounding, which substantially restrict comparability across studies and complicate interpretation (14). First, prior studies have often included heterogeneous populations, with some enrolling all women undergoing vaginal delivery or combining primiparous and multiparous women, even though previous childbirth is itself a major determinant of pelvic floor structure and function; this makes it more difficult to isolate the independent effect of LEA (15). Second, much of the existing literature has focused on symptom-based endpoints, particularly postpartum urinary incontinence, whereas comprehensive evaluation using objective and multimodal pelvic floor measures, such as electrophysiological, biomechanical, or imaging-based parameters, remains relatively limited (16). Third, follow-up timing varies considerably across studies, ranging from the early postpartum period to 6 months after delivery, while the 6–8-week postpartum interval—both clinically feasible for routine follow-up and biologically relevant for early pelvic floor recovery—has not been sufficiently characterized (17). Finally, because LEA is not a randomized exposure and is closely intertwined with labor duration, oxytocin augmentation, instrumental delivery, and other intrapartum management factors, residual confounding remains difficult to eliminate completely even when matching or regression adjustment is applied in observational studies (6). Therefore, further studies are still warranted to clarify the independent association between LEA and postpartum pelvic floor recovery in a more homogeneous population, using early objective pelvic floor assessment together with appropriate adjustment for key peripartum factors (18). In light of the above gaps, the present study was designed to further clarify the association between LEA and early postpartum pelvic floor outcomes in primiparous women with singleton cephalic vaginal deliveries. We used multimodal objective pelvic floor assessments and multivariable regression to evaluate the association between LEA and early postpartum pelvic floor outcomes. Given the observational design and the limited availability of several clinically relevant confounders, the adjusted estimates were interpreted as associations conditional on the measured covariates rather than as causal effects.

## Patients and methods

### Study design and setting

This was a single-center retrospective cohort study conducted at a tertiary maternity center. Clinical and follow-up records of primiparous women who underwent vaginal delivery and returned for postpartum pelvic floor assessment were retrospectively reviewed. The study aimed to investigate whether LEA was associated with early postpartum pelvic floor outcomes in women undergoing vaginal delivery.

This study was approved by the Ethics Committee of Nanjing First Hospital (Approval No: KY20250224-KS-07). All participants provided written informed consent before undergoing the examination. The study complied with the ethical principles of the Declaration of Helsinki.

### Participants

Eligible participants were screened from women who delivered during the study period and had available postpartum follow-up records. The inclusion criteria were as follows: (1) primiparous women; (2) singleton pregnancy; (3) cephalic presentation; (4) vaginal delivery; and (5) availability of postpartum pelvic floor assessment data within the predefined follow-up window.

Women were excluded if they had incomplete key eligibility information, lacked postpartum pelvic floor assessment data required for primary outcome analysis, or could not be classified into the predefined analytic cohorts. After final data cleaning, 200 valid records were included in the overall cohort. Of these, 191 women had available postpartum pelvic floor assessment data and therefore entered the primary outcome cohort. For multivariable regression analyses, a main model-ready cohort of 175 women was constructed by further restricting the primary outcome cohort to cases with complete data for the prespecified covariates included in the adjusted models.

### Exposure definition and group assignment

The exposure of interest was LEA. Participants were classified into the LEA group or the non-LEA group according to whether epidural analgesia was documented as having been administered during labor in the anesthetic and obstetric records. Group assignment reflected actual clinical practice rather than random allocation. Receipt of LEA may therefore have been influenced by clinical indications, evolving labor conditions, maternal preference, and provider-related practice, creating the possibility of systematic differences between the exposure groups. In the present study, LEA was treated as a real-world clinical exposure status indicating receipt of epidural labor analgesia during the delivery process. Detailed characteristics of LEA exposure, including anesthetic regimen, drug concentration, opioid supplementation, timing of epidural placement, maintenance technique, duration of analgesia, and quality of analgesic effect, were not consistently available in the retrospective records and were therefore not incorporated into the exposure definition or subsequent analyses.

### Outcome measures

#### Primary outcomes

The primary outcomes were objective pelvic floor function parameters assessed at the routine postpartum follow-up visit within the predefined 6–8-week window. These parameters were obtained from the structured pelvic floor evaluation dataset and were selected to reflect both neuromuscular function and pelvic floor support. The primary outcomes included type I pelvic floor surface electromyography (sEMG), type II pelvic floor sEMG, bladder neck mobility, posterior bladder angle during the Valsalva maneuver, and levator hiatus area during the Valsalva maneuver. Type I and type II sEMG were used to characterize pelvic floor muscle functional performance, whereas bladder neck mobility, posterior bladder angle, and levator hiatus area were used to reflect structural and supportive changes in the pelvic floor during early postpartum recovery.

#### Secondary outcomes

Secondary outcomes included urinary incontinence, urinary incontinence subtype, pelvic organ prolapse, prolapse stage, and levator avulsion, which were recorded as part of the postpartum pelvic floor follow-up assessment. Urinary incontinence subtype was categorized as stress urinary incontinence, urgency urinary incontinence, or mixed urinary incontinence according to the structured follow-up record. Pelvic organ prolapse and prolapse stage were derived from the postpartum pelvic floor examination record, and levator avulsion was identified from the pelvic floor imaging assessment when available. In addition, peripartum characteristics were analyzed to describe labor progression and delivery-related factors, including duration of labor stages, oxytocin use, artificial rupture of membranes, perineal status, blood loss, postpartum hemorrhage, fetal distress, and amniotic fluid grade.

### Data collection and cohort definitions

Clinical data were extracted from the medical records, anesthetic records, and structured follow-up dataset. Variables collected included baseline maternal characteristics, obstetric characteristics, LEA exposure status, peripartum variables, and postpartum pelvic floor outcomes.

Three analytic datasets were defined before the formal statistical analyses. The overall cohort (*n* = 200) included all valid records after final data cleaning and was used for baseline and peripartum comparisons. The primary outcome cohort (*n* = 191) included women with available postpartum pelvic floor assessment data and was used for comparisons of early postpartum pelvic floor outcomes. The main model-ready cohort (*n* = 175) consisted of women in the primary outcome cohort who also had complete data for all prespecified covariates required for the adjusted regression models. Accordingly, cases with missing primary outcome data were excluded from the primary outcome analyses, and cases with missing values in one or more prespecified covariates were further excluded from the multivariable analyses.

### Statistical analysis

All statistical analyses were performed using SPSS. Continuous variables were first assessed for normality using the Shapiro–Wilk test. Normally distributed variables were presented as mean ± standard deviation (SD) and compared using the independent-samples t test. Non-normally distributed variables were presented as median with interquartile range (IQR) and compared using the Mann–Whitney *U* test. Categorical variables were summarized as number (percentage) and compared using the chi-square test or Fisher's exact test, as appropriate. All tests were two-sided, and a *P* value <0.05 was considered statistically significant.

Baseline characteristics and peripartum variables were analyzed in the overall cohort. Primary and secondary pelvic floor outcomes were analyzed in the primary outcome cohort. Multivariable analyses were performed in the main model-ready cohort. Multivariable regression analyses were performed in the main model-ready cohort. The models adjusted for maternal age, pre-pregnancy body mass index, gestational age, neonatal birth weight, second-stage labor duration, and oxytocin use.

Second-stage labor duration and oxytocin use may not be conventional pre-exposure confounders and may instead lie, at least partly, on a biologically plausible pathway linking LEA with pelvic floor outcomes. Adjustment for these variables could therefore attenuate an association operating through changes in labor progression or intrapartum management. Accordingly, the adjusted estimates were interpreted as associations conditional on the measured baseline and labor-process variables, rather than as estimates of the total effect of LEA or formally identified direct causal effects.

The timing of oxytocin administration relative to LEA initiation was not consistently available in the retrospective records. Therefore, oxytocin use could not be classified definitively as either a pre-exposure confounder or a post-exposure mediator for all participants. Formal mediation analysis was not performed (19).

For continuous pelvic floor outcomes, multiple linear regression models were constructed with LEA as the exposure of interest. For binary outcomes, including urinary incontinence and pelvic organ prolapse, binary logistic regression models were used. Adjusted *β* coefficients or odds ratios (ORs), 95% confidence intervals (CIs), and *P* values were reported. A complete-case analysis strategy was adopted for the multivariable models. Because this was a retrospective observational cohort study, the sample size was determined by the number of eligible participants available during the predefined study period rather than by a prospective sample size calculation. Therefore, no formal *a priori* sample size estimation was performed.

Rationale for analytical approach: Multivariable regression was selected as the primary adjustment method because the main model-ready cohort included 175 participants and several clinically important binary outcomes occurred infrequently. Covariates were selected *a priori* on the basis of clinical relevance, data completeness, and sample-size considerations, and the number of covariates was intentionally limited to reduce the risk of model overfitting. This approach allowed all complete cases to be retained while estimating associations conditional on the measured covariates.

Propensity score matching, inverse probability of treatment weighting, propensity score covariate adjustment, and propensity score-based sensitivity analyses were considered but were not performed. Matching could have excluded unmatched participants and further reduced the number of outcome events available for analysis. Weighting would have required adequate overlap in the probability of receiving LEA and careful evaluation of potentially extreme or unstable weights. Propensity score covariate adjustment would still have relied on the same measured pre-exposure variables available for conventional regression.

Moreover, several clinically relevant factors were unavailable or incompletely recorded in the retrospective dataset. Propensity score methods would therefore not have resolved the principal limitation of unmeasured residual confounding. Accordingly, multivariable regression was retained as a pragmatic approach for estimating adjusted observational associations rather than causal effects.

Given the observational design, the possibility of residual confounding, and the absence of propensity score-based sensitivity analyses, all adjusted estimates were interpreted as observational associations conditional on the measured covariates rather than as causal effects.

## Results

### Study population

**Figure 1:**
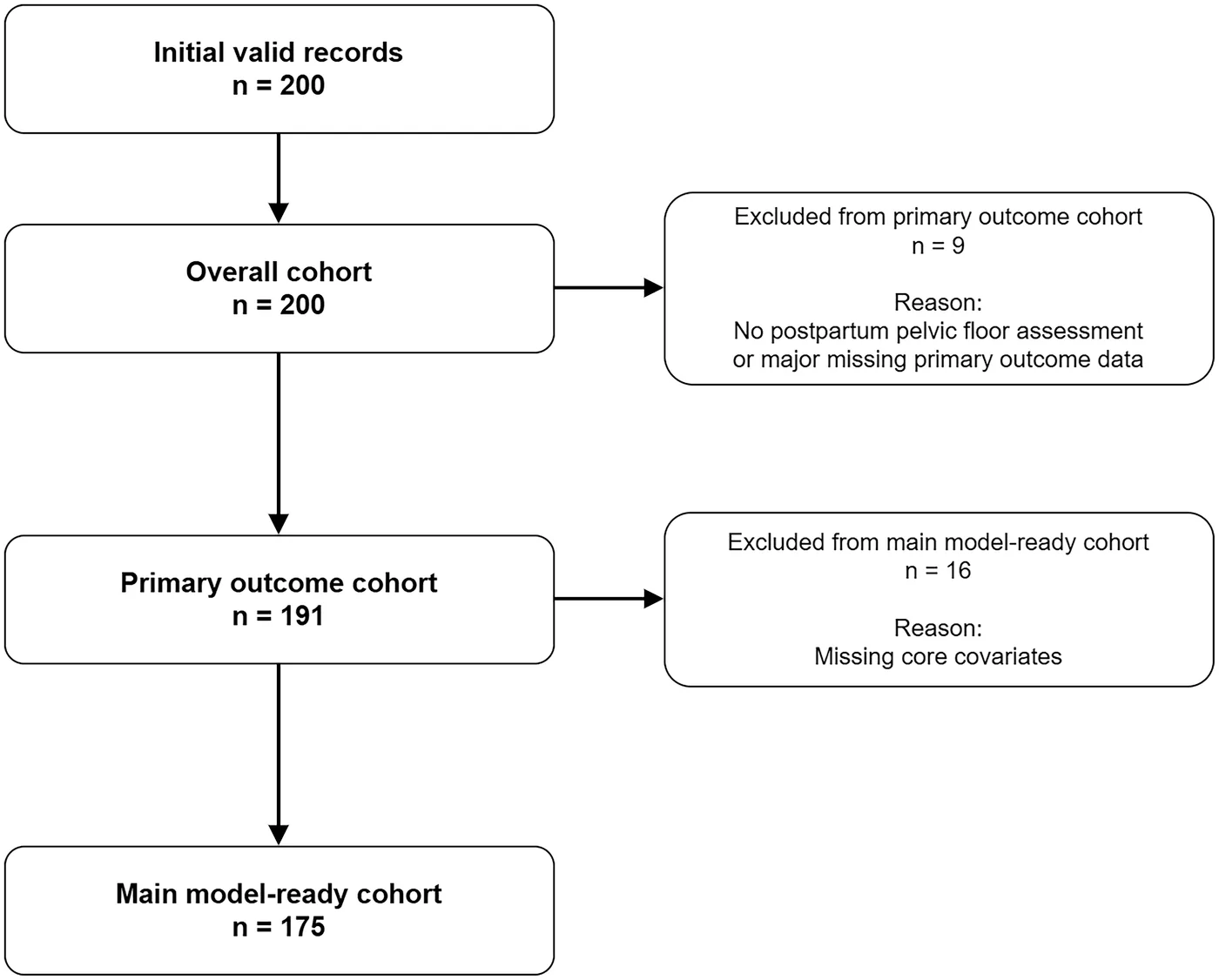
Flowchart of patient selection and analytic cohorts. After final data cleaning, 200 valid records were included in the overall cohort. Of these, 191 women with available postpartum pelvic floor assessment data entered the primary outcome cohort. A further 16 women were excluded from the adjusted analyses because of missing data in one or more prespecified covariates, resulting in a main model-ready cohort of 175 participants.

A total of 200 valid records were included in the overall cohort after final data cleaning. Among these, 9 cases were excluded from the primary outcome cohort because of unavailable postpartum pelvic floor assessment data or major missing primary outcome information, leaving 191 participants for the primary outcome analyses. An additional 16 cases were excluded from the main model-ready cohort because of missing values in one or more prespecified covariates required for the adjusted models, resulting in 175 participants for the multivariable regression analyses. The patient selection process and analytic cohort construction are shown in Figure 1.

### Baseline characteristics

Baseline characteristics of the overall cohort are summarized in Table 1. No statistically significant differences were observed between the LEA group (*n* = 100) and the non-LEA group (*n* = 100) in maternal age, gestational age, pre-pregnancy body mass index, gestational weight gain, or neonatal birth weight. Specifically, maternal age was 29.15 (26.60, 31.20) years in the LEA group and 27.90 (25.58, 30.62) years in the non-LEA group (*P* = 0.077). Gestational age at delivery was 39.41 ± 0.76 weeks and 39.34 ± 0.82 weeks, respectively (*P* = 0.508), while pre-pregnancy body mass index was 22.35 ± 2.73 kg/m^2^ and 22.59 ± 2.83 kg/m^2^ (*P* = 0.539). Gestational weight gain was 13.40 (11.60, 15.30) kg in the LEA group and 14.35 (11.72, 16.60) kg in the non-LEA group (*P* = 0.129), and neonatal birth weight was 3,276.60 ± 335.55 g vs. 3,339.16 ± 338.21 g (*P* = 0.191).

**Table 1:** Baseline characteristics of the overall cohort.

Characteristic	LEA group (*n* = 100)	Non-LEA group (*n* = 100)	*P* value
Maternal age, years	29.15 (26.60, 31.20)	27.90 (25.58, 30.62)	0.077
Gestational age, weeks	39.41 ± 0.76	39.34 ± 0.82	0.508
Pre-pregnancy BMI, kg/m^2^	22.35 ± 2.73	22.59 ± 2.83	0.539
Gestational weight gain, kg	13.40 (11.60, 15.30)	14.35 (11.72, 16.60)	0.129
Neonatal birth weight, g	3,276.60 ± 335.55	3,339.16 ± 338.21	0.191
Macrosomia, *n*/*N* (%)	1/100 (1.00)	4/100 (4.00)	0.369
Gestational diabetes mellitus, *n*/*N* (%)	9/100 (9.00)	8/100 (8.00)	0.809
Hypertensive disorders of pregnancy, *n*/*N* (%)	3/100 (3.00)	7/100 (7.00)	0.331
Anemia during pregnancy, *n*/*N* (%)	12/100 (12.00)	13/100 (13.00)	0.872

Continuous variables are presented as mean ± SD or median (IQR) according to distribution. Percentages were calculated based on the number of non-missing observations for each variable. Pre-pregnancy BMI was available in 94 LEA cases and 97 non-LEA cases. Gestational weight gain was available in 99 LEA cases and 98 non-LEA cases.

Likewise, the prevalence of macrosomia, gestational diabetes mellitus, hypertensive disorders of pregnancy, and anemia during pregnancy did not differ significantly between groups (all *P* > 0.05), indicating that no statistically significant differences were identified in the measured baseline variables.

### Peripartum characteristics

Peripartum characteristics are shown in Table 2. Compared with the non-LEA group, the LEA group had a significantly longer first stage of labor (612.45 ± 190.15 vs. 548.44 ± 187.36 min, *P* = 0.017), second stage of labor (84.69 ± 42.08 vs. 71.45 ± 32.63 min, *P* = 0.014), third stage of labor (10.99 ± 3.74 vs. 9.45 ± 3.54 min, *P* = 0.003), and total labor duration (708.13 ± 197.64 vs. 629.34 ± 185.63 min, *P* = 0.004).

**Table 2:** Peripartum characteristics of the overall cohort.

Characteristic	LEA group (*n* = 100)	Non-LEA group (*n* = 100)	*P* value
First-stage labor, min	612.45 ± 190.15	548.44 ± 187.36	0.017
Second-stage labor, min	84.69 ± 42.08	71.45 ± 32.63	0.014
Third-stage labor, min	10.99 ± 3.74	9.45 ± 3.54	0.003
Total labor duration, min	708.13 ± 197.64	629.34 ± 185.63	0.004
Intrapartum blood loss, mL	236.00 (178.00, 291.00)	219.50 (150.50, 288.00)	0.715
24-h blood loss, mL	375.42 ± 99.41	358.56 ± 92.00	0.226
Oxytocin use, *n*/*N* (%)	36/97 (37.11)	19/95 (20.0)	0.014
Artificial rupture of membranes, *n*/*N* (%)	47/96 (48.96)	38/97 (39.18)	0.221
Perineal status, *n*/*N* (%)			0.817
0°	6/100 (6.0)	5/100 (5.0)	
1°	58/100 (58.0)	53/100 (53.0)	
2°	26/100 (26.0)	32/100 (32.0)	
3°	10/100 (10.0)	10/100 (10.0)	
Postpartum hemorrhage, *n*/*N* (%)	11/97 (11.34)	7/94 (7.44)	0.501
Fetal distress, *n*/*N* (%)	5/100 (5.0)	5/100 (5.0)	1.000
Amniotic fluid grade, *n*/*N* (%)			0.660
0	78/100 (78.0)	80/100 (80.0)	
1	6/100 (6.0)	9/100 (9.0)	
2	5/100 (5.0)	3/100 (3.0)	
3	11/100 (11.0)	8/100 (8.0)	

Continuous variables are presented as mean ± SD or median (IQR) according to distribution. Percentages were calculated based on the number of non-missing observations for each variable. Intrapartum blood loss and 24-h blood loss were available in 97 LEA cases and 94 non-LEA cases. Oxytocin use was available in 97 LEA cases and 95 non-LEA cases. Artificial rupture of membranes was available in 96 LEA cases and 97 non-LEA cases.

In addition, oxytocin use was significantly more frequent in the LEA group than in the non-LEA group [36/97 (37.11%) vs. 19/95 (20.00%), *P* = 0.014]. However, no statistically significant differences were observed between groups in intrapartum blood loss, 24-h blood loss, artificial rupture of membranes, perineal status, postpartum hemorrhage, fetal distress, or amniotic fluid grade (all *P* > 0.05).

### Early postpartum pelvic floor outcomes

Early postpartum pelvic floor outcomes assessed during the routine 6–8-week postpartum pelvic floor follow-up are summarized in Table 3. No statistically significant differences were found between the LEA and non-LEA groups in any of the continuous objective pelvic floor parameters. Type I pelvic floor electromyography was 25.86 ± 4.54 *μ*V in the LEA group and 26.25 ± 4.77 μV in the non-LEA group (*P* = 0.568), while type II electromyography was 33.17 ± 6.26 μV and 33.54 ± 5.77 μV, respectively (*P* = 0.669). Bladder neck mobility was 26.16 ± 4.86 mm in the LEA group and 26.20 ± 4.44 mm in the non-LEA group (*P* = 0.960). Likewise, no significant differences were observed in the posterior bladder angle during the Valsalva maneuver (129.68 ± 10.74° vs. 131.53 ± 11.14°, *P* = 0.245) or levator hiatus area during the Valsalva maneuver (18.65 ± 3.64 vs. 18.85 ± 3.80 cm^2^, *P* = 0.702).

**Table 3:** Early postpartum pelvic floor outcomes in the primary outcome cohort.

Outcome	LEA group (*n* = 94)	Non-LEA group (*n* = 97)	*P* value
Type I pelvic floor EMG, μV	25.86 ± 4.54	26.25 ± 4.77	0.568
Type II pelvic floor EMG, μV	33.17 ± 6.26	33.54 ± 5.77	0.669
Bladder neck mobility, mm	26.16 ± 4.86	26.20 ± 4.44	0.960
Posterior bladder angle,°	129.68 ± 10.74	131.53 ± 11.14	0.245
Levator hiatus area, cm^2^	18.65 ± 3.64	18.85 ± 3.80	0.702
Levator avulsion, *n* (%)	1 (1.06)	2 (2.06)	1.000
Urinary incontinence, *n* (%)	28 (29.79)	24 (24.74)	0.535
Urinary incontinence subtype, *n* (%)			0.861
Mixed	5	3	
SUI	20	18	
UUI	3	3	
Pelvic organ prolapse, *n* (%)	18 (19.15)	12 (12.37)	0.277
POP stage, *n* (%)			0.274
0	76 (80.85)	85 (87.63)	
1	12 (12.77)	9 (9.28)	
2	5 (5.32)	1 (1.03)	
3	1 (1.06)	2 (2.06)	

EMG indicates electromyography; SUI, stress urinary incontinence; UUI, urgency urinary incontinence; POP, pelvic organ prolapse. Percentages were calculated based on the number of observations available for each outcome category. These analyses represent unadjusted between-group comparisons.

Similarly, no significant between-group differences were found in levator avulsion, urinary incontinence, urinary incontinence subtype, pelvic organ prolapse, or prolapse stage (all *P* > 0.05).

### Multivariable analyses

Multivariable regression analyses were performed in the main model-ready cohort (*n* = 175), and the results are presented in Table 4. After adjustment for the measured maternal and peripartum covariates, including second-stage labor duration and oxytocin use, no statistically significant association was observed between LEA and any continuous pelvic floor outcome. Specifically, no significant associations were observed for type I pelvic floor electromyography (*B* = 0.006, 95% CI: −1.494 to 1.505, *P* = 0.994), type II electromyography (*B* = −0.838, 95% CI: −2.822 to 1.146, *P* = 0.405), bladder neck mobility (*B* = −0.362, 95% CI: −1.847 to 1.123, *P* = 0.631), posterior bladder angle during the Valsalva maneuver (*B* = −2.225, 95% CI: −5.731 to 1.282, *P* = 0.212), or levator hiatus area during the Valsalva maneuver (*B* = 0.385, 95% CI: −0.775 to 1.545, *P* = 0.513).

**Table 4:** Multivariable regression analyses for early postpartum pelvic floor outcomes.

Outcome	Model	Effect estimate	95% CI	*P* value
Type I pelvic floor EMG	Linear regression	*B* = 0.006	−1.494 to 1.505	0.994
Type II pelvic floor EMG	Linear regression	*B* = −0.838	−2.822 to 1.146	0.405
Bladder neck mobility	Linear regression	*B* = −0.362	−1.847 to 1.123	0.631
Posterior bladder angle	Linear regression	*B* = −2.225	−5.731 to 1.282	0.212
Levator hiatus area	Linear regression	*B* = 0.385	−0.775 to 1.545	0.513
Urinary incontinence	Binary logistic regression	OR = 1.305	0.629 to 2.710	0.475
Pelvic organ prolapse	Binary logistic regression	OR = 1.771	0.717 to 4.373	0.216

Models were adjusted for maternal age, pre-pregnancy body mass index, gestational age, neonatal birth weight, second-stage labor duration, and oxytocin use. The analyses were performed in the main model-ready cohort (*n* = 175). Because several binary outcomes had relatively low event numbers, particularly pelvic organ prolapse and levator avulsion, the precision of some estimates was limited and confidence intervals should be interpreted cautiously.

**Figure 2:**
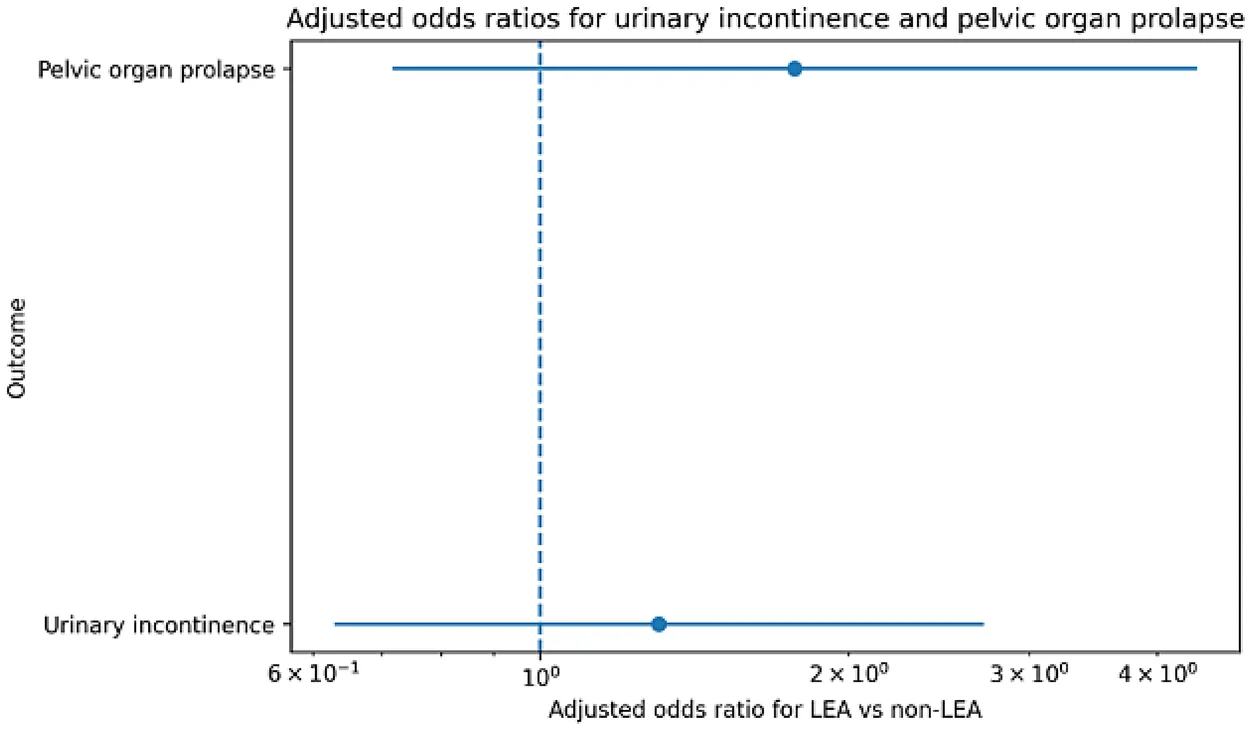
Adjusted odds ratios for urinary incontinence and pelvic organ prolapse associated with LEA. Models were adjusted for maternal age, pre-pregnancy body mass index, gestational age, neonatal birth weight, second-stage labor duration, and oxytocin use. Because second-stage labor duration and oxytocin use may act as labor-process variables, the estimates should be interpreted as conditional associations. In addition, limited event numbers for some outcomes may result in imprecise estimates.

Likewise, binary logistic regression did not identify statistically significant adjusted associations between LEA and urinary incontinence (OR = 1.305, 95% CI: 0.629–2.710, *P* = 0.475) or pelvic organ prolapse (OR = 1.771, 95% CI: 0.717–4.373, *P* = 0.216). Adjusted ORs for urinary incontinence and pelvic organ prolapse are presented in Figure 2.

Overall, no statistically significant between-group differences were observed in the unadjusted pelvic floor outcome comparisons, and no statistically significant associations were observed in the multivariable models conditional on the measured baseline and intrapartum variables. Because second-stage labor duration and oxytocin use may lie on the exposure–outcome pathway, the adjusted estimates should not be interpreted as representing the total effect of LEA. Given the limited number of events for some binary outcomes, particularly pelvic organ prolapse and levator avulsion, the absence of statistical significance should be interpreted as the absence of a statistically detectable association within the available sample rather than definitive evidence of no association.

## Discussion

In this cohort of primiparous women undergoing vaginal delivery, LEA was associated with longer first-, second-, and third-stage labor, longer total labor duration, and more frequent oxytocin use, suggesting that its primary influence may occur within the labor process. However, no statistically detectable association was observed between LEA and early postpartum pelvic floor outcomes after adjustment for measured covariates. Recent cohort evidence similarly demonstrated that epidural analgesia was associated with prolonged labor duration among women undergoing vaginal delivery, without significant adverse maternal–neonatal outcomes; however, pelvic floor outcomes were not evaluated (20). These findings should be interpreted as conditional observational associations rather than evidence excluding causal or pathway-mediated effects, because LEA exposure was determined by clinical practice rather than random allocation.

Overall, our findings are consistent with the systematic review by Cardoso et al., which found insufficient evidence for a consistent association between LEA and postpartum urinary incontinence (4), but differ from Xu et al., who reported an association between LEA and early postpartum stress urinary incontinence in a propensity score-matched cohort (6). These discrepancies may reflect differences in study populations, outcome definitions, follow-up timing, and approaches to confounding control. Our findings support a more nuanced framework in which the primary influence of LEA may occur at the level of the labor process rather than being directly translated into measurable pelvic floor impairment within the early postpartum period. Recent prospective studies have similarly reported longer first- and second-stage labor among women receiving epidural analgesia, supporting an association between LEA and altered labor dynamics (1, 21).

The association between LEA and prolonged labor observed in our study is consistent with previous prospective evidence (1, 21). Du et al. similarly reported longer first- and second-stage labor among women receiving epidural analgesia, supporting a reproducible relationship between LEA and altered labor dynamics (6). Mechanistically, LEA-related prolongation of labor may involve reduced pain perception, altered maternal pushing dynamics, changes in pelvic floor–abdominal pressure coordination, and accompanying intrapartum management strategies such as oxytocin augmentation. Therefore, LEA should be considered not only as an anesthetic exposure but also as part of a broader labor management context (22).

The interpretation of second-stage labor duration and oxytocin use requires particular caution because these variables may represent mediators rather than conventional confounders. If LEA influences pelvic floor outcomes partly through altered labor progression or intrapartum management, adjustment for these intermediate variables may attenuate pathway-related associations and should not be interpreted as estimating the total effect of LEA (19).

Therefore, the present multivariable estimates should be understood as associations conditional on measured labor-process variables rather than causal effects. Because the timing of oxytocin administration relative to LEA initiation was not consistently available, we were unable to distinguish mediation from confounding or formally evaluate direct and indirect effects. The absence of a statistically detectable association in this conditional model therefore does not exclude potential effects operating through labor-related pathways. Future longitudinal studies incorporating detailed temporal information and mediation approaches are needed to clarify whether observed associations arise from LEA itself, labor progression, or related intrapartum management factors (23, 24).

The absence of statistically detectable differences in objective pelvic floor measures and symptom-based outcomes between LEA and non-LEA groups suggests that LEA may not be associated with measurable additional pelvic floor impairment during the early postpartum period. This finding is consistent with the overall direction of evidence from the prospective study by Du et al. and the systematic review by Cardoso et al., which indicate that although LEA may influence peripartum process indicators, its association with postpartum pelvic floor dysfunction remains inconsistent (10, 15). Vaginal delivery itself is likely to remain a major determinant of pelvic floor structural and neuromuscular loading, while any additional influence of LEA may be modest or mediated through labor-related pathways rather than sufficient to produce detectable differences at 6–8 weeks postpartum. The early postpartum period represents a dynamic phase of pelvic floor remodeling, during which subtle differences may not yet be stabilized or may be obscured by individual recovery variability (25). The combined use of electrophysiological and imaging-based assessments strengthens the interpretation of these findings, as objective measures provide complementary information regarding neuromuscular function and pelvic support. Although bladder neck mobility and levator hiatus dimensions have been associated with postpartum pelvic floor symptoms (26), differences in these parameters may not necessarily emerge across all obstetric exposures within the early recovery window.

Symptom-based outcomes showed a similar pattern, with no statistically detectable differences between LEA and non-LEA groups after adjustmen (8, 9). These findings are broadly consistent with the systematic review by Cardoso et al., which concluded that current evidence is insufficient to support a consistent association between LEA and postpartum urinary incontinence (13). However, they differ from Xu et al., who reported an increased risk of early postpartum stress urinary incontinence among women receiving LEA (4), suggesting that symptom-based outcomes may be particularly sensitive to differences in study populations, follow-up timing, and analytical approaches. Several factors may contribute to these discrepancies. First, urinary incontinence and prolapse symptoms during the early postpartum period may reflect ongoing tissue remodeling, hormonal changes, and behavioral adaptation rather than established long-term dysfunction (27).

Second, symptom-based outcomes are influenced by subjective perception, reporting thresholds, and postpartum activity patterns, which may result in greater variability than objective pelvic floor measurements (28). In addition, the relatively low event numbers for pelvic organ prolapse and levator avulsion limited statistical precision; therefore, the present findings should be interpreted as no statistically detectable association within the available sample rather than definitive evidence excluding a potential effect. Longer-term studies integrating symptom trajectories with objective pelvic floor assessments are needed to clarify whether early postpartum differences persist or resolve over time.

Beyond the empirical findings, our study provides a more nuanced framework for interpreting the relationship between LEA and postpartum pelvic floor recovery. Rather than considering LEA as a direct determinant of pelvic floor dysfunction, our findings suggest that its influence may occur primarily through labor-related processes and intrapartum management pathways. In this cohort, LEA was associated with longer labor duration and greater oxytocin use but was not associated with worse objective or symptom-based pelvic floor outcomes at 6–8 weeks postpartum. Therefore, the relationship between LEA and pelvic floor recovery may be better understood as a multistep pathway in which labor-related changes are further modified by additional factors, including delivery-related mechanical load, perineal trauma, baseline maternal characteristics, and recovery dynamics (29). This framework may help explain the inconsistent findings across previous studies, as different investigations may capture different points along the same biological pathway rather than identical outcomes (30). Future research integrating repeated measurements, detailed intrapartum characterization, and mediation approaches will be important to clarify whether and in whom LEA-related labor changes translate into clinically meaningful pelvic floor outcomes.

From a clinical perspective, these findings suggest that LEA should not be avoided solely because of concerns regarding short-term pelvic floor deterioration.4 Among primiparous women undergoing vaginal delivery, clinical decisions regarding LEA should consider the balance between analgesic benefits, maternal preference, labor progression, and overall obstetric context rather than assuming that LEA inevitably increases pelvic floor injury risk. The present results also highlight that clinical attention may need to shift from the presence of LEA itself toward optimization of modifiable intrapartum factors, including second-stage labor management and rational oxytocin use, because labor-related pathways may represent more relevant targets for intervention. Furthermore, postpartum pelvic floor screening should remain focused on overall obstetric risk rather than exposure to a specific analgesic technique alone. Objective electrophysiological and imaging-based assessments may provide additional information for identifying women who require closer follow-up and rehabilitation (11, 31). For patient counseling, clinicians should communicate that current evidence supports an effect of LEA on labor characteristics, whereas its independent association with short-term pelvic floor dysfunction remains uncertain.

Several methodological strengths of the present study should be highlighted. First, the study included a relatively homogeneous cohort of primiparous women with singleton, cephalic, vaginal deliveries, reducing heterogeneity related to previous obstetric history and delivery characteristics. Second, rather than relying solely on symptom-based outcomes, this study incorporated multimodal electrophysiological and imaging assessments of pelvic floor function and support, providing a more comprehensive evaluation of early postpartum recovery. Third, the 6–8-week postpartum assessment represents a clinically relevant early recovery window, allowing evaluation of pelvic floor restoration during a period of active remodeling. Finally, multivariable regression models incorporating key maternal and peripartum variables strengthened the interpretation of the observed associations beyond simple between-group comparisons.

### Limitations

First, this was a single-center retrospective cohort study, and LEA exposure reflected clinical practice, maternal preference, and evolving labor conditions rather than random allocation. Therefore, selection bias, indication bias, and residual confounding cannot be excluded. Although regression models adjusted for several measured maternal and peripartum variables, important factors including LEA indications, fetal position, fetal head characteristics, operative vaginal delivery, perineal trauma, pushing characteristics, intrapartum position, and provider-related practice differences were unavailable or incompletely recorded. These factors may influence postpartum pelvic floor recovery, as prolonged second-stage labor and levator ani injury have been associated with poorer pelvic floor outcomes (25), while operative vaginal delivery, fetal head characteristics, and obstetric anal sphincter injury have been linked to childbirth-related levator injury (32, 33). Provider-related variation may also contribute to unmeasured heterogeneity because assisted vaginal delivery depends on operator experience and clinical practice patterns (34). Although adjustment methods can reduce measured confounding, they cannot fully eliminate bias from unavailable variables, as illustrated by previous propensity score analyses showing persistent imbalance in clinically relevant obstetric factors (14). In addition, LEA was analyzed as a binary exposure because detailed epidural characteristics, including timing, anesthetic regimen, dosage, opioid supplementation, maintenance strategy, duration, and analgesic effectiveness, were not consistently available. Therefore, the present study cannot determine whether specific epidural exposure patterns are associated with differential pelvic floor outcomes.

Second, second-stage labor duration and oxytocin use may represent mediators rather than conventional confounders because they may lie on the pathway between LEA and pelvic floor outcomes. Adjustment for these variables may attenuate pathway-related associations; therefore, the present estimates should be interpreted as associations conditional on measured labor-process variables rather than total or causal effects of LEA. Because detailed temporal information regarding oxytocin administration relative to LEA initiation was unavailable, formal mediation analysis could not be performed (19). In addition, no antepartum pelvic floor assessment was available, follow-up was limited to 6–8 weeks postpartum, and event numbers for outcomes such as pelvic organ prolapse and levator avulsion were relatively low, limiting assessment of long-term recovery and precision for infrequent outcomes. Larger prospective studies with repeated measurements and detailed temporal characterization of intrapartum interventions are needed.

Third, propensity score matching, inverse probability of treatment weighting, propensity score adjustment, and related sensitivity analyses were not performed. Although these approaches may improve comparability between exposure groups based on measured covariates, their validity remains dependent on the completeness of available pre-exposure information. In the present study, the moderate sample size, limited available covariates, and absence of several clinically relevant obstetric factors reduced the expected reliability of additional propensity score analyses. Therefore, the findings should be interpreted as adjusted observational associations rather than causal estimates or evidence that LEA and non-LEA groups were fully exchangeable.

Future studies should adopt multicenter prospective designs incorporating antepartum baseline assessment, repeated postpartum measurements, detailed LEA characterization, and temporal information on intrapartum interventions. Such approaches may clarify whether labor-mediated pathways contribute to pelvic floor recovery and allow more appropriate evaluation of direct and indirect effect (23).

## Conclusion

In this single-center retrospective cohort of primiparous women undergoing vaginal delivery, LEA was associated with longer labor duration and more frequent oxytocin use, whereas no statistically detectable association with worse early postpartum pelvic floor outcomes was identified after adjustment for the measured covariates. Within the binary LEA exposure framework evaluated in this study, these findings suggest that LEA was not associated with measurable additional pelvic floor impairment during the early postpartum recovery period. However, given the observational design, potential residual confounding, incomplete exposure characterization, and limited precision for infrequent outcomes, these findings should be interpreted cautiously. Larger prospective multicenter studies with detailed intrapartum characterization and longer follow-up are warranted.

## Data Availability

The original contributions presented in the study are included in the article/Supplementary Material, further inquiries can be directed to the corresponding author.
